# A right eye amaurosis patient with congenital absence of the internal carotid artery: A care compliant case report

**DOI:** 10.1097/MD.0000000000017779

**Published:** 2019-11-01

**Authors:** Yun-Long Ding, Jia-Li Niu, Li Ma, Zhi-Qun Gu, Ting-Ting Zhai, Yan Liu

**Affiliations:** a Department of Neurology; b Department of Clinical Pharmacy, Jingjiang People's Hospital, the Seventh Affiliated Hospital of Yangzhou University, Jiangsu; c Department of Neurology, Shaoxing Second Hospital, the Second Affiliated Hospital of Shaoxing University, Zhejiang, China.

**Keywords:** collateral pathway, congenital absence, digital subtraction angiography, internal carotid artery

## Abstract

**Rationale:**

Absence or hypoplasia of the internal carotid artery (ICA) are rare developmental anomalies. Usually, patients with ICA agenesis are asymptomatic due to collateral circulation, but they may present with seizures, headache, or transient ischemic attack. We report a patient with right ICA absence in whom “paroxysmal right eye amaurosis” was the main symptom.

**Patient concerns:**

A 76-year-old male patient suffered from “paroxysmal right eye amaurosis for 3 years”. Three years prior, the patient had suffered sudden one-minute right eye amaurosis without any obvious cause. The attack reoccurred 1–2 times/year until one week before admission when he experienced two sudden right eye amaurosis.

**Diagnosis:**

Congenital absence of the right ICA was diagnosed. In this patient with congenital absence of the right ICA, the ipsilateral anterior cerebral artery (ACA) was compensated by the anterior communicating artery (ACOM), and the ipsilateral middle cerebral artery (MCA) emerged from the carotid siphon of the contralateral ICA.

**Interventions:**

The patient was given antiplatelet treatment consisting of aspirin and atorvastatin after admission and instructed to maintain the treatment after discharge.

**Outcomes:**

No symptom onset was observed during follow-up.

**Lessons:**

Here, we report the patient's clinical manifestations and imaging findings and analyze the cause of the condition to provide a clinical reference for the study of congenital absence of the ICA.

## 1 Introduction

The internal carotid artery (ICA) is an important blood vessel in the anterior circulation of the brain. During ICA development, abnormal changes can occur in each segment. The ICA rarely exhibits developmental abnormalities,^[1]^ which occur in only 0.01% of the population.^[2]^ Patients with ICA agenesis may present with asymptomatic conditions, transient ischemic attack (TIA), subarachnoid hemorrhage (SAH), developmental delays, hypopituitarism, or developmental malformations in multiple organs.^[3]^ We present a case report of a patient with “paroxysmal right eye amaurosis” as the main symptom who was diagnosed with “right-sided ICA agenesis”. In this patient with congenital absence of the right ICA, the ipsilateral anterior cerebral artery (ACA) was compensated by the anterior communicating artery (ACOM), and the ipsilateral middle cerebral artery (MCA) emerged from the carotid siphon of the contralateral ICA. We report the patient's clinical manifestations and imaging findings and analyze the cause of the condition to provide a clinical reference for the study of congenital absence of the ICA.

## 2 Case presentation

A 76-year-old man was admitted to the hospital on June 13, 2018, due to “paroxysmal right eye amaurosis for 3 years”. Three years prior, the patient suffered a sudden one-minute episode of amaurosis in the right eye without any obvious cause. The attack reoccurred 1 to 2 times/year. The patient experienced sudden amaurosis in the right eye twice in one week before being admitted to the hospital for further treatment. He had a 5-year history of hypertension, and his maximum blood pressure (BP) reached 190/100 mmHg. He reported long-term use of amlodipine (5 mg/day), smoking 400 cigarettes/year for 30 years, and denied any history of alcohol abuse. A physical examination showed a BP of 134/80 mmHg, clear consciousness, verbal communication, bilateral pupil diameters of 2.0 mm, sensitivity to light reflection, normal eye movements, normal limb muscle strength and tension, bilateral sacral reflex symmetry, and no signs of meningeal irritation; no bilateral pathology was identified.

**Figure 1:**
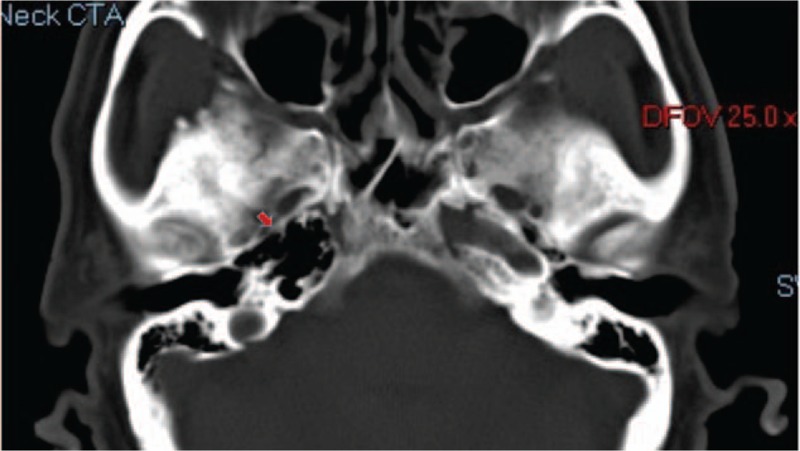
CT image of the skull base showing the complete absence of the right carotid canal with extension of the pneumatization of the right petrous bone (arrowhead), consistent with congenital ICA agenesis.

**Figure 2:**
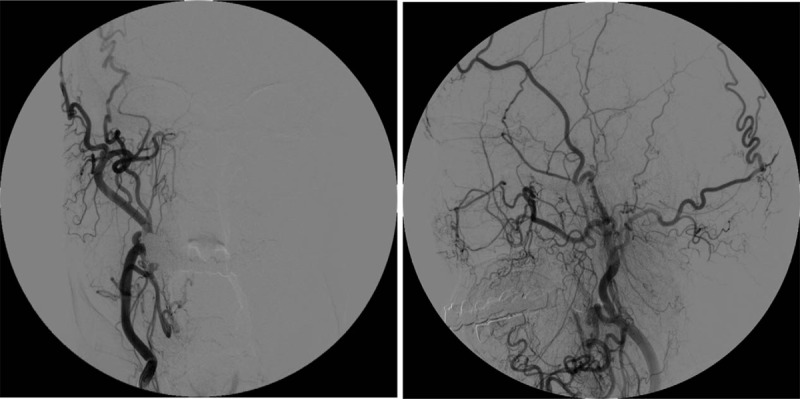
DSA - right carotid artery angiography. High-pressure syringe setting: 8 ml/s; total volume: 12 ml. The right ICA was absent, the ipsilateral ophthalmic artery was compensated by the middle meningeal artery, and no compensation for the external carotid artery to the brain was observed. DSA = digital subtraction angiography, ICA = internal carotid artery.

**Figure 3:**
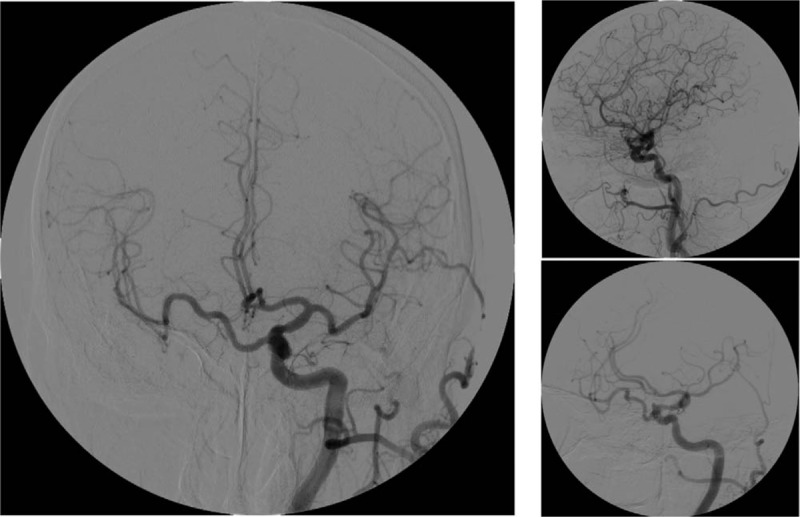
DSA - left carotid artery angiography. High-pressure syringe setting: 8 ml/s; total volume: 12 ml. No abnormalities were observed in the left MCA and ACA. The left ICA supplied the contralateral ACA through the ACOM. The ipsilateral MCA emerged from the left carotid siphon. ACA = anterior cerebral artery, ACOM = anterior communicating artery, DSA = digital subtraction angiography, ICA = internal carotid artery, MCA = middle cerebral artery.

**Figure 4:**
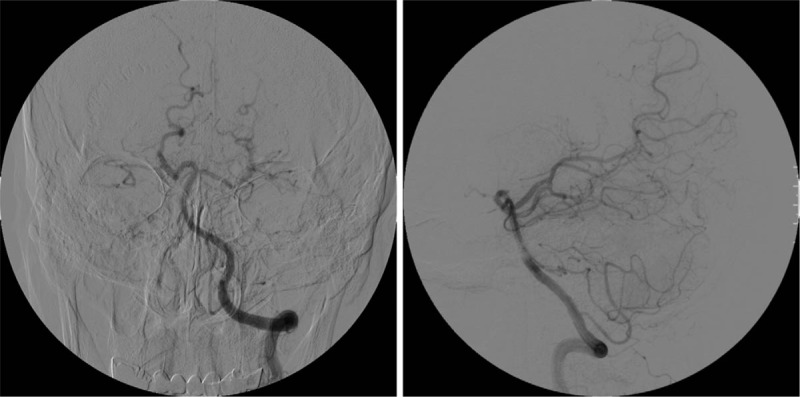
DSA - left vertebral angiography. High-pressure syringe setting: 4 ml/s; total volume: 6 ml. No collateral flow was observed from the posterior circulation to the anterior circulation. DSA = digital subtraction angiography.

After admission, the ophthalmology consultation examination showed no abnormalities in the eyeballs, fundus, or visual field. No notable abnormalities were observed on a head MRI. Brain computed tomography angiography (CTA) showed that the right carotid artery was absent. CT imaging of the skull base showed the complete absence of the right carotid canal (Fig. 1). Digital subtraction angiography (DSA) showed that the right ICA was absent, the ipsilateral MCA emerged from the contralateral carotid siphon, and the ipsilateral ACA was compensated by the contralateral ICA (Figs. 2–4). The discharge diagnoses were: 1. congenital absence of the right ICA and 2. hypertension (level 3, very high-risk).

The patient was given antiplatelet treatment consisting of aspirin and atorvastatin after admission and instructed to maintain the treatment after discharge. No symptom onset was observed during follow-up.

## 3 Discussion

The ICA rarely exhibits developmental abnormalities. It is currently believed that the embryonic environment or factors in it influence embryonic vascular development.^[4]^ In the case of congenital absence of the ICA, little ossification of the carotid artery occurs or the carotid artery does not develop. A head CT scan of this patient showed that the right carotid artery was hypoplastic; therefore, development of the ipsilateral ICA did not occur.

Six types of collateral pathway development can occur in the congenital absence of the ICA.^[3]^ Type A involves the unilateral absence of the ICA and is associated with collateral circulation to the ipsilateral ACA through a patent ACOM and to the ipsilateral MCA from the posterior circulation through a hypertrophied posterior communicating artery (PCOM) or unilateral absence of the ICA associated with collateral circulation to the ipsilateral ACA and MCA from the posterior circulation through a hypertrophied PCOM. In Type B, the ipsilateral ACA and MCA are supplied across a patent ACOM in the presence of the unilateral absence of the ICA. In Type C, there is a bilateral absence of the ICA with supply to the anterior circulation provided through carotid-vertebrobasilar anastomoses generally accomplished through hypertrophy of the PCOM. In Type D, there is a unilateral absence of the ICA with intercavernous communication to the ipsilateral carotid siphon from the contralateral cavernous ICA. In Type E, diminutive ACAs are supplied by bilateral hypoplastic ICAs or unilateral absence of the ICA with contralateral hypoplastic ICA, and the MCAs are supplied by hypertrophied PCOMs. Finally, in Type F, there is collateral flow to the ICA through transcranial anastomoses from the internal maxillary branches of the external carotid artery (ECA) system.

DSA of this patient showed that the right ICA was absent, and the A1 segment of the left ICA supplied the contralateral ICA through the ACA, but the contralateral A1 segment of the ACA was absent. Additionally, vertebral artery angiography showed no collateral flow from the posterior circulation to the anterior circulation, similar to what is observed in Type D. However, Type D exhibits unilateral absence of the ICA with intercavernous communication to the ipsilateral carotid siphon from the contralateral cavernous ICA, and angiography in this patient did not show the development of a right ICA siphon. Given the diameter of the blood vessels and the diameter of the tube, it appeared that the right MCA, which emerged from the contralateral ICA siphon, maintained the blood flow. In contrast to type A and B cases, in this patient, the MCA emerged directly from the contralateral ICA siphon, resulting in an extension of the MCA.

Patients with congenital absence of the ICA have no symptoms in the early stage, but an incomplete circle of Willis is thought to be closely related to ischemic cerebrovascular disease.^[5]^ With increasing age, cerebral TIAs and even cerebral infarction may occur due to cerebral hypoperfusion. In addition, cerebral hemodynamic changes may lead to an aneurysm.^[6,7]^ This patient had no history of intracranial aneurysm and had no clear hypoperfusion, potentially as a result of the benefits of his unique compensation pathway. Type A is associated with collateral circulation to the ipsilateral MCA from the posterior circulation through a hypertrophied PCOM artery, while a blocked PCOM artery reduces the incidence of posterior communicating hemodynamic-related aneurysms. In type B, the ipsilateral MCA is supplied across a patient's ACOM, and the patient's ACOM therefore only needs to supply blood to the right ACA; this may reduce the probability of anterior communicating hemodynamic-related aneurysms. Moreover, the right MCA, which originates directly from the contralateral ICA, also sustains the blood supply well, reducing the risk of intracranial ischemia. However, it is undeniable that the pressure in the left ICA will also increase. The resulting ischemia in the left ICA will cause infarction of the bilateral cerebral hemisphere, which may be fatal.

Congenital absence of the ICA leads to abnormal development of the ophthalmic artery, but reliable recurrent compensation can prevent ischemic events. At present, the ophthalmic artery compensation observed in patients with congenital absence of the ICA is derived from the ipsilateral middle meningeal artery or MCA.^[1]^ The right ophthalmic artery of this patient was compensated by the ipsilateral middle meningeal artery. He had no symptoms during the early stage, and we speculate that as he ages, arteriosclerosis and other factors could cause the compensated blood flow to be insufficient to sustain perfusion, resulting in TIAs. However, further basic and clinical research is still required.

Currently, there are no guidelines regarding the management of congenital absence of the ICA. For patients with aneurysm or ruptured aneurysm, the indications for interventional embolization or craniotomy should be assessed, and patients with vascular stenosis or ischemic symptoms could take antiplatelet and statin drugs to prevent ischemic events.^[8]^ Hou reported a patient with pontine infarction and congenital hypoplasia of the left ICA who was given anti-platelet aggregation, statin and other drugs for secondary prevention and achieved a good outcome.^[9]^ This patient was admitted to the hospital due to “paroxysmal right eye amaurosis for 3 years”. DSA and CT examination confirmed the absence of the right ICA. Considering that amaurosis was associated with the onset of ischemic events in the ocular artery blood supply area after the exclusion of eye diseases, we therefore provided the patient with medications for ischemic events. This patient did not show symptoms of sputum after taking aspirin and atorvastatin, suggesting that the treatment may have been effective, but further follow-up and large-sample studies are needed. For patients with congenital absence of the ICA in which a single ICA supplies blood to the bilateral anterior circulation, close monitoring of the ICA should be performed to prevent the occurrence of ischemic events and avoid catastrophic bilateral cerebral hemisphere infarction.

## Author contributions

**Conceptualization:** Yun-Long Ding, Jia-Li Niu, Li Ma, Ting-Ting Zhai, Yan Liu.

**Data curation:** Yun-Long Ding, Jia-Li Niu, Zhi-Qun Gu.

**Investigation:** Yun-Long Ding.

**Supervision:** Li Ma, Ting-Ting Zhai, Yan Liu.

**Writing – original draft:** Yun-Long Ding, Jia-Li Niu, Zhi-Qun Gu.

**Writing – review & editing:** Yun-Long Ding, Li Ma, Ting-Ting Zhai, Yan Liu.

Yan Liu orcid: 0000-0002-3367-7590.
